# Current aspects of cardiac resynchronisation therapy

**DOI:** 10.1007/s12471-015-0779-1

**Published:** 2015-12-07

**Authors:** M. Meine, M.J.M. Cramer, E.E. van der Wall

**Affiliations:** 1 Department of Cardiology, Division Heart & Lungs, University Medical Center Utrecht, Heidelberglaan 100, 3584 CX Utrecht, The Netherlands; 2 Netherlands Society of Cardiology/Holland Heart House, Utrecht, The Netherlands

In 1993, the cardiac surgeon Bakker et al. [1] introduced biventricular pacing as a novel method to treat heart failure by synchronous stimulation of the right and left ventricle. After this first-in-man implantation, the rapid development of transvenous left ventricular (LV) leads and the implementation of biventricular pacing in implantable cardioverter/defibrillators have established cardiac resynchronisation therapy (CRT) as a standard treatment of heart failure with systolic LV dysfunction and broad QRS complexes. Although the milestone trials have proven the benefit of CRT (reduction in mortality and morbidity, reverse remodelling, improvement of LV function), the prediction of CRT response still remains a challenge [2–6]. Because of the high number of CRT non-responders, especially in patients with unspecific widening of the QRS complex, class I indication for CRT was restricted to heart failure patients with typical left bundle branch block (LBBB) in the European Heart Rhythm Association guidelines update of 2013. Two-dimensional echocardiography is the most widely used noninvasive method for the evaluation of LV function and assessment of reverse remodelling after CRT; however it has as yet failed to play an additional role in determining the indication for CRT [7]. Furthermore, even though mechanical dyssynchrony was thought to be present using echocardiographic parameters, CRT was harmful in those patients with narrow QRS complexes [8]. In more than 20 years of experience with CRT-related issues, we have deepened our knowledge about indication, implantation, evaluation, and optimisation of CRT. With the rapid development of the CRT technology new challenges arise.

This special issue of the Netherlands Heart Journal elucidates current aspects of cardiac resynchronisation therapy. Wiegerinck et al. [9] describe the pathophysiological relationship between LBBB and dyssynchronous mechanical activation which creates LV dysfunction by ‘wasted work’. The worsening of LV function causes remodelling by neurohumoral activation and asymmetric hypertrophy. The fact that these remodelling processes are caused by changes in the regulation of gene expression raises the question whether genetic predisposition can also play a role in CRT response. Possible answers to this question are discussed in the review article by Lahrouchi and Bezzina [10]. They found different expressions of genes encoding components of Ca^2 +^ handling, β-adrenergic receptors, contractile proteins, and myocardial natriuretic peptide pre- and post-CRT. It remains unclear whether these changes in gene expression are truly induced by CRT itself or whether they are the result of improvement in LV function. Because of the complexity of the phenotype ‘CRT responder’ and the intricate underlying genetic architecture, large studies will be necessary to identify genetic factors associated with volumetric CRT response.

Versteeg et al. [11] showed in a prospective study of 139 CRT patients that patient-reported outcome assessed prior to CRT independently identifies poor survival and hospitalisation. It seems logical to assess patient-perceived symptoms of heart failure, functional limitations, and quality of life routinely before CRT implantation and during follow-up to improve their management. In the accompanying editorial comment ‘Patients predict their own outcome’, Kronborg and Nielsen [12] appeal for implementation of patient-reported outcomes when taking care of these heart failure patients who need cardiac implantable devices. The question remains whether interventions performed to improve patient-reported health status also influence the long-term outcome of CRT patients. Heart failure symptoms classified by NYHA and exercise capacity were the primary endpoints of the multicentre randomised trials at the beginning of the CRT era [2–6]. Later on, the evaluation of hospitalisation rate, survival, and reverse remodelling became more important to convince the healthcare authorities about the value of CRT. Reverse remodelling can be assessed by echocardiography with its limitations of imaging quality and interobserver variability. Van Everdingen et al. [13] critically discuss the role of echocardiography in their review article. The importance of measuring dyssynchrony to select patients for CRT seemed to increase with every new published single-centre study until the multicentre PROSPECT trial brought disappointment to imaging cardiologists [7]. Ghani et al. [14] showed in their prospective observational single-centre study of 297 CRT patients that echocardiographic assessment of mechanical dyssynchrony (apical rocking) predicted CRT super-responders with a good long-term prognosis. Van ’t Sant et al. [15] showed that the amount of reverse remodelling can be a surrogate marker for long-term outcome only in patients with non-ischaemic cardiomyopathy. In ischaemic cardiomyopathy CRT may resolve LV dyssynchrony, however coronary artery disease still determines the patient’s prognosis.

One important, underestimated factor that influences CRT response is LV lead placement. Imaging techniques such as cardiac MRI tagging or deformation echocardiography can detect the segment of latest mechanical activation; however prior to CRT implantation the exact coronary venous anatomy is usually not known. So why don’t we measure the electrical delay in the side branches of the coronary sinus where the LV pacing lead will be implanted? Such an optimal lead position at the free wall of the LV may be more important in patients with ischaemic cardiomyopathy and non-typical LBBB. Van Stipdonk et al. [16] describe a new method to find instantaneously delayed LV lateral wall activation by electroanatomical mapping in the coronary veins during CRT implantation. Especially for patients with non-LBBB QRS widening this new method facilitates optimal LV lead placement and may improve response to CRT [17]. In comparison with LV lead optimisation, little is known about the contribution of right ventricular (RV) pacing to CRT. Expert opinion about the necessity of RV pacing for CRT can be divided in the following two directions: (1) Biventricular pacing to optimise diastolic function and (2) LV pacing with fusion of intrinsic right bundle branch depolarisation to obtain optimal ventricular synchronicity of depolarisation. In the contribution by Wu et al. [18] it was shown that in an invasive observational study of 41 CRT patients biventricular pacing improved haemodynamic response in comparison to only LV pacing in patients with impaired RV function. On the other hand, RV pacing ‘only’ can be harmful, especially for patients with impaired LV function. For these patients who develop heart failure during conventional pacemaker therapy an upgrade to CRT is indicated. However, the milestone trials about CRT included only ‘de novo’ CRT implants and the common opinion is that CRT upgrade procedures are more difficult and complicated compared with ‘de novo’ implants. In a retrospective single-centre study Ter Horst et al. [19] showed that CRT upgrades were not more complex nor associated with more complications than ‘de novo’ CRT implantations. Notably, these results can only be applied to a tertiary centre with a high patient throughput and three highly experienced cardiologists who performed the procedures, as De Voogt pointed out [20]. The decision of an upgrade procedure to a more complex system has to be carefully considered. An ‘upgrade’ to endocardial LV pacing might be much more complex than an upgrade from a conventional device to a CRT. The Eindhoven group has extensive experience with LV endocardial lead placement. They showed that the benefit of LV endocardial pacing is based on the absence of phrenic nerve stimulation and the free choice of LV lead placement independently of having a coronary vein at the target region. The most significant haemodynamic improvement of CRT with LV endocardial pacing was in the region of LV delayed electrical activation [21]. These data must motivate us to put all our efforts into placing the LV lead in the target region. Special catheter techniques for ‘getting the LV lead to the right spot’ are described by Jackson and Steen [22].

Several current aspects of cardiac resynchronisation therapy are highlighted in this special CRT issue. These reviews and original articles can give us new insights for daily clinical practice and provide inspiration for new research projects to further improve heart failure therapy with implantable cardiac devices for our patients.
